# Practical Chemistry for Students and Physicians—Chapter Seventh

**Published:** 1884-01

**Authors:** 


### Editorial.

### PRACTICAL CHEMISTRY FOR STUDENTS AND PHYSICIANS.—CHAPTER SEVENTH.

Continued from December REGISTER, Vol. III, Page 87.

The next element of the halogen group to be considered, is bromine. Symbol Br.; atomic weight, 80; quantivalence, 1, 5 and 7; spc. gravity, 3.187; density of vapor, 80.

*Occurrence.*—Bromine is found in the waters of the sea and of certain mineral springs, in combination chiefly with the metals sodium and magnesia. It was in the waters of the mediterranean sea, that in 1826, Balard, a French chemist, discovered it. He detected it in the bittern or mother liquor, in the process of extracting from those waters their crystallizable salt. Bromine is also found in a few minerals. There is an ore of silver, embolite, a chlorobromide of silver found in Chili, not less abundant than horn silver—AgCl. Balard named his new discovery "bromine," from the Greek *bromos*—a stench—on account of its disgusting odor.

*Preparation of bromine.*—It is obtained from the residual liquor of salt works, from which the sodium chloride, common salt, sodium sulphate and magnesia sulphate, all less soluble than the salts of bromine, NaBr KBr and $MgBr_2$ have been crystalized by evaporation and removed.

Chlorine being more active chemically than bromine, is capable of detaching it from its compounds. Advantage is taken of this fact, in the next step of the process. Chlorine gas is, now, passed through the mother liquor, till it assumes an orange color, from the diffusion of the free bromine. This is the reaction: Na Br $_2 xCl_2 = 2NaClxBr_2$. But how is the bromine to be separated from the water? It can not be done by evaporation, for the bromine is, itself, very volatile. It is effected by the superior adhesive attraction or solvent powder of ether. Ether is now shaken briskly with the bromine water; the bromine leaving the water, mixes with the ether, and this, finally, having little affinity for the water, rises and settles upon the surface in a beautiful orange colored mixture.

How, secondly, is the bromine separated from the ether? This is easily done by bringing into service the superior chemical attraction of a solu ion of caustic potash. The ether bromine layer is decanted or carefully poured off, and shaken with a solution of potash, which instantly removes the bromine from the ether, leaving the latter to rise, as before, to the surface, but now pure. The reaction is as follows: $6KHOxBr_6 = 5 KBrxKBrO_3 x3H_2 O$ The solution containing the bromate and bromide of potassium, having been evaporated to dryness, is heated to discompose the bromate and change it into bromide: $KBrO_3 = KBrxO_3$. Thirdly, from the KBr, the bromine is separated by distilling it with manganese deoxide, $MnO_2$ and sulphuric acid. The hydrogen of the $H_2 SO_4$ is oxidized at the expense of the $MnO_2$, leaving nothing to prevent the isolation of the bromine $2KBr xMnO_2 x2H_2 SO_4 = K_2 SO_4 xMnSO_4 x2H_2 OxBr_2$.

